# The attitude of contemporary Iranian directors and screenwriters toward patients with mental disorders in comparison with general population

**DOI:** 10.1186/s40359-024-01550-x

**Published:** 2024-02-15

**Authors:** Kiandokht Kamalinejad, Seved Vahid Shariat, Negin Eissazade, Mohammadreza Shalbafan

**Affiliations:** 1 Mental Health Research Center, Psychosocial Health Research Institute, Department of Psychiatry, School of Medicine, Iran University of Medical Sciences, Tehran, Iran; 2 Brain and Cognition Clinic, Institute for Cognitive Sciences Studies, Tehran, Iran

**Keywords:** Stigma, Mental disorders, Directors, Screenwriters, General Population

## Abstract

**Background:**

Mental disorders are accountable for 16% of global disability-adjusted life years (DALYs). Therefore, accessible, cost-effective interventions are needed to help provide preventive and therapeutic options. As directors and screenwriters can reach a great audience, they can use their platform to either promote stigma or educate the public with the correct definition and conception of mental disorders. Therefore, we aimed to measure the stigmatizing attitude of contemporary Iranian directors and screenwriters toward patients with mental disorders in comparison with a general population group.

**Methods:**

In this comparative study, we included 72 directors and screenwriters between 18 and 65 years of age with a minimum involvement in at least one movie/television show, and 72 age and educationmatched controls. We collected the demographic data of the participants, and used the Persian version of the Level of Contact Report (LCR) to measure their familiarity with mental disorders, and used the Persian version of Social Distance Scale (SDS) and Dangerousness Scale (DS) to measure their attitude toward them.

**Results:**

Compared to the general population group, directors and screenwriters had significantly lower SDS (12.51 ± 3.8 vs. 13.65 ± 3.73) and DS (12.51 ± 3.8 vs. 13.65 ± 3.73) scores (*P* < 0.001), indicating a more positive attitude toward patients with mental disorders. Familiarity with mental disorders was not significantly different between the groups. Female sex was associated with a more negative attitude among the directors and screenwriters group. Additionally, among the SDS items, ‘*How would you feel about someone with severe mental disorder marrying your children?*’ and ‘*How would you feel about someone with severe mental disorder taking care of your children for a couple of hours?*’ received the most negative feedback in both groups. And among the DS items, ‘*there should be a law forbidding a former mental patient the right to obtain a hunting license*’ received the most negative feedback in both groups.

**Conclusions:**

Iranian contemporary directors and screenwriters had a more positive attitude toward patients with mental disorders, compared to general population. Due to this relatively positive attitude, this group of artists can potentially contribute to anti-stigma initiatives by offering educational materials and resources, promoting mental health care, and improving access to mental health care.

## Background

Mental disorders account for 16% of the global disability-adjusted life years (DALYs). Subsequently, there is a pressing demand for accessible cost-effective preventive, supportive, and therapeutic strategies [1]. A significant number of patients with mental disorders do not seek mental healthcare, due to the discriminatory attitudes of the public towards mental disorders, commonly referred to as stigma. Stigma results in social exclusion, self-stigma, isolation, and ultimately, reduced quality of life [2, 3].

Cinema and television hold significant power over shaping the perceptions and attitudes of the public on various issues, with mental health being no exception. At the same time, the artists working in this field, are influenced by societal attitudes, as the reception of their work not only mirrors current perceptions but also adds to the ongoing dialogue around mental health. Therefore, cinema and television can be adopted as tools for either reinforcing stigma or reducing it. Nevertheless, individuals with mental disorders are frequently portrayed as violent, unpredictable, and inferior, perpetuating negative stereotypes. The exaggeration and sensationalization of mental disorders in media, contribute to the persistence of misconceptions. Furthermore, portrayals of psychiatrists and psychologists often lack empathy, reflecting them as ineffective, or at times, even harmful in their treatment methods [4–8].

Stigma against mental disorders in the Middle East is significantly affected by sociocultural factors, creating barriers to open discussions and impeding access to mental healthcare [9, 10]. However, due to anti-stigma interventions, there has been an increasing awareness of mental health issues and a greater willingness to seek treatment in recent years [9]. As filmmakers can reach a great audience, they can use their platform to educate the public with the correct definition and conception of mental disorders. Therefore, we aimed to evaluate the stigmatizing attitude of contemporary Iranian directors and screenwriters toward patients with mental disorders in comparison with a general population group.

## Methods

### Design and participants

This comparative study was conducted between February 2021 and August 2022. We reached out to directors and screenwriters via their official social media accounts, with inclusion criteria specifying an age range of 18–65 years and a minimum involvement in at least one movie or television show. Controls, matched for age and education, were volunteer social media users and university staff, excluding hospital staff.

### Tools

In our survey, we collected the demographic information of the participants, along with the Persian versions the Level of Contact Report (LCR), Social Distance Scale (SDS), and Dangerousness Scale (DS) [11–14].

The LCR is a tool for assessing familiarity with mental disorders. It consists of twelve situations with varying degrees of intimacy with patients with mental disorders, listed in increasing order of familiarity. Participants are asked to score each item from 1 (*I have never observed a person with mental illness*) to 4 (*I have a severe mental illness*). Higher scores indicate more familiarity with mental disorders. If more than one category applied for a respondent, we selected the one with the highest familiarity. The Cronbach’s alpha coefficient for the Persian version of this questionnaire was reported as 0.427 in the coercion structure and between 0.75 and 0.91 in other structures [11, 13].

The SDS is used to assess the attitude toward patients with mental disorders. It presents a patient with a severe mental disorder and asks the participants to rate their level of comfort with interacting with them in seven different hypothetical scenarios, on a scale of zero to three. Total score ranges from 0 to 21, with higher scores indicating greater discomfort and desire for distance. We used the Persian version of this scale, for which Cronbach’s alpha coefficient has been reported as 0.96, retest coefficient as 0.88, and content validity as 0.77 [12, 14].

The DS also measures the attitude toward patients with mental disorders. It consists of eight questions asking the reaction of the respondent in particular situations involving a patient with a mental disorder. It is scored using a 7-point Likert scale, from ‘completely disagree’ to ‘completely agree.’ Total score ranges from eight to 56, and higher scores indicate higher levels of perceived dangerousness. For the Persian version of this scale, the Cronbach’s alpha coefficient has been reported as 0.92, retest coefficient as 0.89, and content validity as 0.75 [12, 13].

### Statistical analysis

Statistical analyses were conducted using Statistical Package for the Social Sciences (SPSS) software for Windows (version 27, SPSS Inc., Chicago, IL, USA). Descriptive statistics are presented as mean ± standard deviation. Categorical variables were compared using the Chi-square and Fisher’s exact tests. Normality was assessed using the Shapiro-Wilk test. Continuous variables were compared using the Pearson and Spearman tests, along with the independent t-test, and the Mann-Whitney U test. The correlations between LCR, SDS, and DS scores and the categorical variables were assessed using one-way ANOVA. A *p*-value of  0.05 or less was considered statistically significant.

## Results

A total of 144 participants with a mean age of 43.04 ± 10.9 years completed our survey (72 directors and screenwriters and 72 controls), of which 92 (63.9%) were male. The majority of the participants in the directors and screenwriters group were male (*N* = 59, 81.9%). Demographic data of the participants are presented in Table 1.

**Table 1:** Demographic data of the participants (count, %)

	Directors and screenwriters (*N* = 72)	Control group (*N* = 72)	Total (*N* = 144)
**Gender**			
Female	13 (18.1%)	39 (54.2%)	52 (36.1%)
Male	59 (81.9%)	33 (45.8%)	92 (63.9%)
**Positive personal history of mental illness**	2 (2.7%)	2 (2.7%)	4 (2.8%)
**Positive family history of mental illness**	18 (25%)	11 (15.2%)	29 (20.1%)
**Education level**			
High school diploma	2 (2.8%)	2 (2.8%)	4 (2.8%)
Associate degree	5 (6.9%)	5 (6.9%)	10 (6.9%)
Bachelor’s degree	36 (50%)	36 (50%)	72 (50%)
Master’s degree	26 (36.1%)	26 (36.1%)	52 (36.1%)
Ph.D	3 (4.2%)	3 (4.2%)	6 (4.2%)

### LCR

The mean scores of LCR were 10.72 ± 8.09 in the directors and screenwriters group and 9.26 ± 6.3 in the control group. LCR scores were not significantly different between the two groups (*P* = 0.152) (Table 2). Among the participants’ demographic data, only higher number of movies/television shows was associated with higher LCR scores (*P* = 0.014) (Table 3).

**Table 2:** Comparison of the questionnaires’ scores between directors and screenwriters and the control group (Mann-Whitney U-test)

	Directors and screenwriters	Control	*P*-value
LCR	12.18 ± 9.30	9.26 ± 6.36	0.152
DS	32.14 ± 6.50	34.92 ± 7.31	0.017*
SDS	12.51 ± 3.8	13.65 ± 3.73	< 0.001*

*LCR: Level of Contact Report, DS: Dangerousness Scale, SDS: Social Distance Scale*

*Correlation is significant at the 0.05 level

**Table 3:** The *p*-values of correlations between the demographic data and LCR, DS, and SDS scores of the participants

	LCR		DS		SDS	
	**Directors and screenwriters**	**Control**	**Directors and screenwriters**	**Control**	**Directors and screenwriters**	**Control**
Age	0.294	0.688	0.689	0.952	0.119	0.073
Sex	0.656	0.314	0.602	0.792	0.029*	0.468
Positive personal history of mental disorder	0.781	0.614	0.21	0.639	0.412	0.529
Positive family history of mental disorder	0.835	0.415	0.9	0.201	0.939	0.077
Education level	0.31	0.384	0.807	0.411	0.417	0.259
Number of movies/television shows	0.014*	-	0.671	-	0.165	-
Movies/television shows about mental disorders	0.209	-	0.359	-	0.819	-

*LCR: Level of Contact Report, DS: Dangerousness Scale, SDS: Social Distance Scale*

*Correlation is significant at the 0.05 level

Further analysis by linear regression model, revealed that mean LCR score is significantly correlated with the number of movies/television shows (*P* = 0.01), and not with sex (*P* = 0.66), age (*P* = 0.39), education level (*P* = 0.20), movies/television shows about mental disorders (*P* = 0.56), and personal (*P* = 0.30) and family (*P* = 0.91) history of mental disorders.

### SDS

The mean scores of SDS were 12.51 ± 3.8 and 13.65 ± 3.73 in the directors and screenwriters group and control group, respectively. Directors and screenwriters had significantly lower SDS (*P* < 0.001) scores (Table 2).

Higher SDS scores were significantly associated with the female sex in the directors and screenwriter group (*P* = 0.029). Education level, positive personal and family history of mental disorders, and movies/television shows about mental disorders did not have any significant correlations with SDS scores (Table 3). Adjusting the analysis for gender, did not lead to different results.

Among the directors and screenwriters group, ‘*How would you feel about someone with severe mental disorder taking care of your children for a couple of hours?*’ (*N* = 34, 47.2%) and ‘*How would you feel about someone with severe mental disorder marrying your children?*’ (*N* = 32, 44.4%) received the most negative feedback, respectively.

Among the control group, ‘*How would you feel about someone with severe mental disorder marrying your children?*’ (*N* = 45, 62.5%) and ‘*How would you feel about someone with severe mental disorder taking care of your children for a couple of hours?*’ (*N* = 43, 59.7%) received the most negative feedback, respectively.

Further analysis by linear regression model, revealed that SDS scores are only correlated with sex (*P* = 0.013), and not related with age (*P* = 0.18), education level (*P* = 0.96), number of movies/television shows (*P* = 0.74), movies/television shows about mental disorders (*P* = 0.78), and personal (*P* = 0.531) and family (*P* = 0.82) history of mental disorders.

### DS

The mean scores of DS were 12.51 ± 3.8 and 13.65 ± 3.73 in the directors and screenwriters group and controls, respectively. Directors and screenwriters had significantly lower DS (*P* = 0.017) scores (Table 2). The correlations between the DS scores and demographic data of the participants was non-significant (Table 3).

The item ‘*There should be a law forbidding a former mental patient the right to obtain a hunting license*’ received the most negative feedback in both directors and screenwriters group (*N* = 10, 13.9%) and controls (*N* = 18, 25.0%).

In addition, a significant association was found between the SDS and DS scores in both directors and screenwriters group (*P* < 0.001) and controls (*P* < 0.001). LCR scores were significantly associated with both SDS (*P* = 0.013) and DS (*P* = 0.024) scores in the directors and screenwriters group.

Further analysis by linear regression model, revealed that DS scores were significantly correlated with personal history of mental disorder (*P* = 0.003) and number of movies/television shows (*P* = 0.031), and not with sex (*P* = 0.534), age (*P* = 0.10), education level (*P* = 0.56), movies/television shows with mental disorders (*P* = 0.56), and family history of mental disorder (*P* = 0.95).

## Discussion

Compared to general population, directors and screenwriters had significantly lower SDS and DS scores, indicating a more positive attitude toward patients with mental disorders. Female sex was significantly associated with a more negative attitude in the directors and screenwriters group. Familiarity with mental disorders was not significantly different between the groups. Positive personal history of mental disorders was significantly associated with higher DS scores. Additionally, in both groups, ‘*How would you feel about someone with severe mental disorder marrying your children?*’ and ‘*How would you feel about someone with severe mental disorder taking care of your children for a couple of hours?*’ received the most negative feedback among the SDS items, and ‘*There should be a law forbidding a former mental patient the right to obtain a hunting license*’ received the most negative feedback among the DS items.

We found that directors and screenwriters had a more positive attitude toward patients with mental disorders which might be due to artists’ creativity and flexibility, and exposure to cultural diversity. However, in contrast to previous studies, we did not find any association between familiarity with patients with mental disorders and the stigmatizing attitude toward them [15–18] which raises questions about the specific nature of familiarity and previous contact with patients with mental disorder. As suggested by previous studies, encouraging direct, regular and interactive contact with these individuals may allow for a deeper understanding of their experiences and struggles, and challenging preconceived inaccurate negative stereotypes [4, 19, 20].

Female sex was previously reported to be associated with a positive attitude toward patients with mental disorders [21]. However, based on our results, female sex was associated with a greater tendency for social distance among directors and screenwriters. However, the small number of female participants in this group makes this finding inconclusive.

We did not find any significant association between age and stigmatizing attitude. However, previous studies have reported that older age is associated with a more negative attitude towards patients with mental disorders [22].We did not find any significant correlations between education level and stigmatizing attitude. Although, it has been reported that a higher level of education is associated with a more positive attitude toward patients with mental disorders [23, 24].

Positive personal history of mental disorders was significantly associated with a lower level of perceived dangerousness. Similarly, previous studies have reported that a positive personal history of mental disorders is associated with a more positive attitude [24, 25].

We did not find any significant associations between positive family history of mental disorders and stigmatizing attitude. However, previous studies have reported that positive family history of mental disorders is associated with a more negative attitude. However, based on previous studies, perceived dangerousness is significantly lower in individuals with a positive family history of mental disorders [25, 26].

The study of Eissazade et al. (2022) which was conducted to investigate the attitude of Iranian theater artists toward patients with mental disorders, did not report any associations between demographic data and stigmatizing attitude [27]. Moreover, the mean score of SDS (12.51 ± 3.8 vs. 10.67 ± 4.92) and DS (33.53 ± 7.03vs. 28.87 ± 10.291) were higher in our study. However, we investigated different groups of artists with different sample sizes [27].

In the directors and screenwriters group, among the SDS items, ‘*How would you feel about someone with severe mental disorder taking care of your children for a couple of hours? (*similar to the study of Eissazade et al. (2022)), and ‘*How would you feel about someone with severe mental disorder marrying your children?*’ received the most negative feedback. Some concerns exist surrounding the ability of patients with severe mental disorders to ensure children’s safety and welfare, as these disorders can sometimes involve impaired judgment or erratic behavior. However, the most reported child abuse perpetrators are among families or acquaintances [27, 28].

In line with the study of Eissazade et al. (2022), ‘*There should be a law forbidding a former mental patient the right to obtain a hunting license*’ received the most negative feedback among the DS items. Mental disorders can sometimes be associated with impaired cognition or impulsivity, raising apprehensions about the ability to responsibly handle firearms. However, no clear link has been found between violent crimes and mental disorders, without substance use. Also, there has been no substantial report of firearm victims killed by patients with mental disorders in Iran over the past years [27, 29]. Gaining insight into the factors contributing to violence can help with reducing stigma toward these patients.

Anti-stigma programs are needed to raise awareness by offering educational materials and resources, improve access to mental healthcare, and support advocacy efforts to promote mental health policy and legislation. Directors and screenwriters have a significant role in educating the public as they can reach a large and diverse audience, and advocate for policy changes. And therefore, cinema and television can be adopted as powerful tools for reducing the stigma surrounding mental disorders by presenting realistic portrayals and breaking down harmful stereotypes, and subsequently contribute to promoting empathy and social inclusion for patients with mental disorders, and creating safe spaces for these individuals to openly discuss their experiences [30–33].

### Limitations

Our study had limitations such as a small sample size, a cross-sectional design, potential participation bias, self-reporting bias, and a restricted choice of questionnaire. Given the significance and prevalence of mental disorders, future investigations should encompass larger and more diverse samples across various artistic domains to achieve more comprehensive results.

## Conclusions

In conclusion, Iranian contemporary directors and screenwriters had a more positive attitude toward patients with mental disorders compared to the general population. Due to this relatively positive attitude, this group of artists can potentially contribute to anti-stigma initiatives by offering educational materials and resources, promoting mental health care, and improving access to mental health care.

## Data Availability

The dataset used and analyzed during the current study can be shared by the corresponding author upon reasonable request.
